# Left anterior descending artery dissection masquerading as takotsubo syndrome ventriculography: you shall not be fooled

**DOI:** 10.1093/ehjcr/ytae435

**Published:** 2024-08-20

**Authors:** Ana Rita Bello, Rita A Carvalho, Bruno M L Rocha, Pedro Freitas

**Affiliations:** Cardiology Department, Hospital de Santa Cruz, Avenida Professor Doutor Reinaldo dos Santos, Carnaxide, Lisboa 2790-134, Portugal; Cardiology Department, Hospital de Santa Cruz, Avenida Professor Doutor Reinaldo dos Santos, Carnaxide, Lisboa 2790-134, Portugal; Cardiology Department, Hospital de Santa Cruz, Avenida Professor Doutor Reinaldo dos Santos, Carnaxide, Lisboa 2790-134, Portugal; Cardiomyopathy Outpatient Unit, Hospital de Santa Cruz, Carnaxide, Portugal; Advanced Heart Failure and Heart Transplantation, Hospital de Santa Cruz, Carnaxide, Portugal; Cardiology Department, Hospital de Santa Cruz, Avenida Professor Doutor Reinaldo dos Santos, Carnaxide, Lisboa 2790-134, Portugal; Cardiac Imaging Department, Hospital de Santa Cruz, Carnaxide, Portugal

## Case presentation

**Figure 1:**
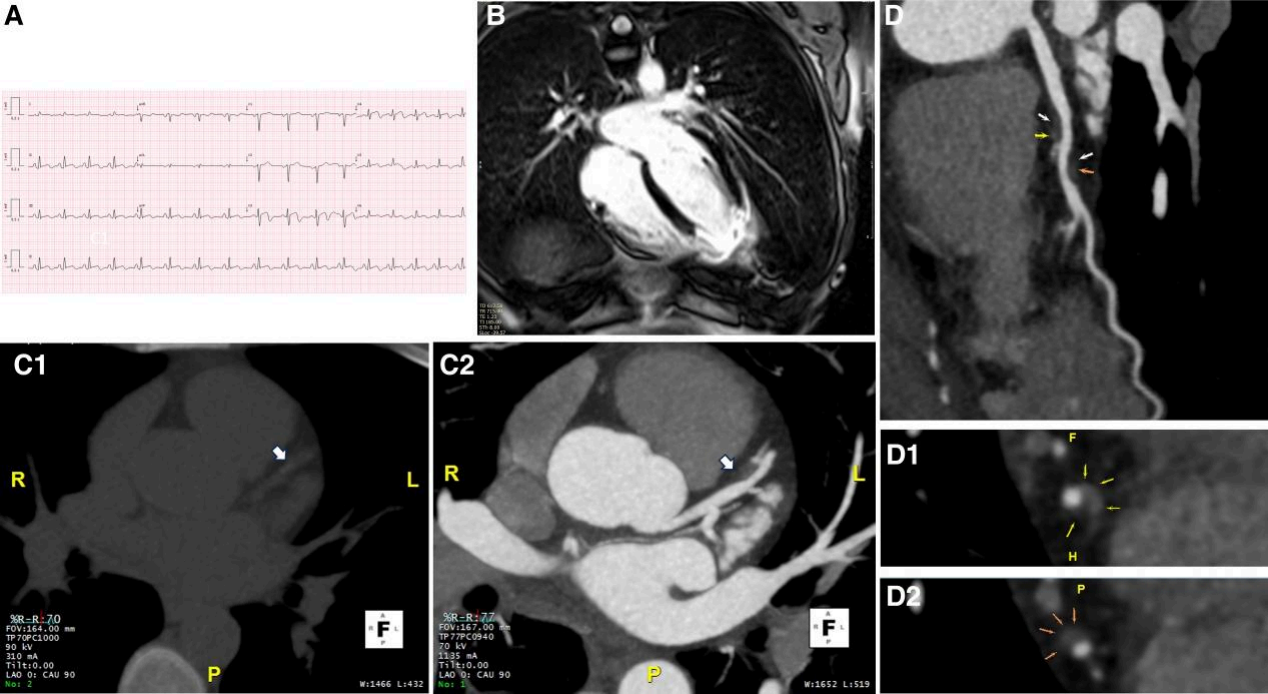
(*A*) Twelve-lead electrocardiogram at admission, showing ST elevation and T wave inversion from V3 to V6. There are no pathological Q waves. Corrected QT interval was normal (427 ms, calculated with Fridericia formula). (*B*) Cardiac magnetic resonance showing apical transmural late gadolinium enhancement, indicating an ischaemic lesion in usual left anterior descending artery territory. (*C*) Coronary computed tomography (MIP reconstruction; 5 mm) with evidence of mural haematoma in mid-left anterior descending artery consistent with spontaneous coronary artery dissection. (*C1*) Pre-contrast acquisition with an area of increased density (90 UH) adjacent to the medium left anterior descending artery (arrow). (*C2*) Post-contrast acquisition demonstrating intramural haematoma in the same area (arrow). (*D*) Curved-multiplanar reformat of the left anterior descending artery, exhibiting mural haematoma. (*D1* and *D2*) Multiplanar reformat of the left anterior descending artery in short axis, depicting the extent of the haematoma proximally (D1, arrows) and distally (D2, arrows).

A 42-year-old woman visited the emergency department due to intermittent chest pain for 24 h, after a stressful event. Her medical history was remarkable for smoking habits. She presented with sinus tachycardia (100 b.p.m.) and a blood pressure of 100/60 mmHg. At admission, the 12-lead electrocardiogram (*Figure 1A*) revealed anterior ST-segment elevation with T wave inversion; QT interval was normal. Transthoracic echocardiogram was remarkable for a non-dilated left ventricle with apical akinesia and preserved left ventricular (LV) ejection fraction. She was transferred to the cath lab for emergent coronary angiography with the hypothesis of an ST-segment elevation myocardial infarction. At first glance, there were no evident signs of epicardial obstructive coronary artery disease. A ventriculography was performed demonstrating LV apical ballooning and hypercontractility of the basal segments (see Supplementary material online, *Video S1*). The patient was admitted with a suspicion of classical takotsubo syndrome (TTS), and no intra-coronary imaging was performed considering the typical clinical setting and findings. Initial cardiac biomarkers were remarkable for a high-sensitivity troponin T (665 ng/L) and N-terminal prohormone of brain natrurietic peptide (NT-proBNP) (791 pg/mL). To continue the workup of myocardial infarction with non-obstructive coronary artery (MINOCA), a cardiac magnetic resonance (CMR) was performed, exhibiting subendocardial late gadolinium enhancement (ischaemic pattern) at the level of the LV true apex (see Supplementary material online, *Video S2A* and *B*; *Figure 1B*). At coronary angiography revision, a suspicion for left anterior descending artery (LAD) spontaneous coronary artery dissection (SCAD) (see Supplementary material online, *Video S3A–D*) motivated a coronary computed tomography (CT) angiography, which further corroborated the diagnosis (*Figure 1C1* and *C2*). A new coronary angiogram was not performed given favourable clinical evolution and to prevent complications from LAD manipulation in the setting SCAD. After being started on bisoprolol and aspirin, she was discharged at day 5 post-admission, after complete symptom cessation and a sustained decrease in cardiac biomarkers. At 3-month follow-up, the patient remained asymptomatic, and 3-month CT re-evaluation showed similar findings, suggesting persistent dissection.

This case highlights the challenges of the workup of MINOCA and the importance of multimodal imaging for individualized therapy, particularly in patients at low risk for atherosclerotic disease presenting with a seemingly typical ventriculography for TTS.^1,2^

## Supplementary Material

ytae435_Supplementary_Data

## Data Availability

The data underlying this article are available in the article and in its online supplementary material.

## Supplementary material

**Informed consent and ethics statement:** The patient verbally consented to the publication of her medical case in a peer-reviewed medical journal. The authors of this article, who actively participated in the decision process and management, obtained the written informed consent from the patient, in accordance with the COPE guidelines.

The authors declare that the figures and videos in the article, including supplementary material, do not allow the identification of the patient. Dates were omitted to comply with confidentiality. This case report was exempt from the ethics’ board approval.

**Funding:** None declared.
